# Non-parametric deconvolution using Bézier curves for quantification of cerebral perfusion in dynamic susceptibility contrast MRI

**DOI:** 10.1007/s10334-021-00995-0

**Published:** 2022-01-13

**Authors:** Arthur Chakwizira, André Ahlgren, Linda Knutsson, Ronnie Wirestam

**Affiliations:** 1 Department of Medical Radiation Physics, Skåne University Hospital, Lund University, 22185 Lund, Sweden; 2 AMRA Medical AB, Linköping, Sweden; 3 Russell H. Morgan Department of Radiology and Radiological Science, Johns Hopkins University School of Medicine, Baltimore, MD USA; 4 F.M. Kirby Research Center for Functional Brain Imaging, Kennedy Krieger Institute, Baltimore, MD USA

**Keywords:** Magnetic resonance imaging, Algorithms, Cerebral circulation, Brain/blood supply, Computer simulation

## Abstract

**Objective:**

Deconvolution is an ill-posed inverse problem that tends to yield non-physiological residue functions *R*(*t*) in dynamic susceptibility contrast magnetic resonance imaging (DSC-MRI). In this study, the use of Bézier curves is proposed for obtaining physiologically reasonable residue functions in perfusion MRI.

**Materials and methods:**

Cubic Bézier curves were employed, ensuring *R*(0) = 1, bounded-input, bounded-output stability and a non-negative monotonically decreasing solution, resulting in 5 parameters to be optimized. Bézier deconvolution (BzD), implemented in a Bayesian framework, was tested by simulation under realistic conditions, including effects of arterial delay and dispersion. BzD was also applied to DSC-MRI data from a healthy volunteer.

**Results:**

Bézier deconvolution showed robustness to different underlying residue function shapes. Accurate perfusion estimates were observed, except for boxcar residue functions at low signal-to-noise ratio. BzD involving corrections for delay, dispersion, and delay with dispersion generally returned accurate results, except for some degree of cerebral blood flow (CBF) overestimation at low levels of each effect. Maps of mean transit time and delay were markedly different between BzD and block-circulant singular value decomposition (oSVD) deconvolution.

**Discussion:**

A novel DSC-MRI deconvolution method based on Bézier curves was implemented and evaluated. BzD produced physiologically plausible impulse response, without spurious oscillations, with generally less CBF underestimation than oSVD.

**Supplementary Information:**

The online version contains supplementary material available at 10.1007/s10334-021-00995-0.

## Introduction

The commonly used perfusion-related parameters cerebral blood flow (CBF), cerebral blood volume (CBV) and mean transit time (MTT) are of value in the assessment of, for example, ischemic threshold level and ischemic penumbra in stroke [1, 2], as well as for characterization and grading of brain tumors [3]. In this context, dynamic susceptibility contrast magnetic resonance imaging (DSC-MRI) is a well-established technique for assessment of parameters related to cerebral perfusion and microvasculature [4, 5].

DSC-MRI is based on the injection of contrast agent (CA), with subsequent measurement of the MRI signal loss during the CA bolus passage through the tissue of interest. Quantification of perfusion parameters from DSC-MRI data is based on general tracer kinetic theory, assuming that the measured tissue concentration time curve (CTC), calculated from the signal curve, can be expressed as the convolution of the arterial input function (AIF) and the tissue residue function scaled with CBF (i.e., the tissue impulse response function) [4–6]. Inference of CBF and MTT from DSC-MRI, thus, requires accurate voxel-wise deconvolution of the observed CTC with the measured AIF. Furthermore, the shape of the tissue residue function reflects microvascular tracer retention and capillary velocity distribution [7, 8], and the residue function may contain information about flow heterogeneity and tissue oxygen extraction [9].

Deconvolution is, in general, an ill-posed inverse problem where slight alterations in the CTC may translate to a dramatic influence on the residue function [8]. Furthermore, effects on arterial bolus delay and dispersion occurring between the site of the AIF measurement and the arterial inlet of the tissue of interest need to be considered in the deconvolution procedure [10–12]. Ideally, the applied AIF should reflect the arterial input to each tissue voxel being analyzed, but the AIF is typically sampled in a major artery (such as the middle cerebral artery or its branches) to minimize partial volume effects [13]. Numerous deconvolution techniques have previously been proposed in the literature [e.g. 5, 7, 8, 14, 15]. Fourier techniques are insensitive to bolus delay but have been shown to poorly estimate high flow rates coupled with noise [5]. The algebraic, model-independent singular value decomposition (SVD) technique was shown to be a robust alternative [5], and Wu et al. later introduced the delay-insensitive block-circulant SVD (oSVD) version [15]. Apart from CBF underestimation in many practical situations, a major weakness of SVD-based deconvolution methods is the induction of non-physiological oscillations in the obtained residue function [5]. Various regularization techniques have been adopted to reduce these oscillations, for example, SVD with a fixed threshold for the singular values, oSVD incorporating an oscillation index [15] and Tikhonov regularization [16].

Mouridsen et al. proposed a vascular model (VM) which produces smooth residue functions and is believed to provide more accurate perfusion estimates than SVD-based methods [8]. The VM was implemented in a Bayesian framework modeling tracer transit times with a gamma probability distribution function. Two parameters (one for shape and another for scale) describe the distribution. Consequently, the resulting residue functions belong to a restricted family of shapes, which may impose restrictions on the applicability and versatility of the VM approach.

Mehndiratta et al. introduced a non-parametric control point interpolation (CPI) deconvolution method, implemented in a Bayesian framework [7]. With the aid of control points, CPI parallels the flexibility in residue function shape that is obtained with non-parametric techniques. Imposing constraints on the control points allows discrimination against non-physiological residue functions. However, CPI exhibits some degree of oscillatory behavior and CBF overestimation in the case of a boxcar residue function [7]. A general cause for concern is that the requirement of the residue function to pass through all control points leads to a large number of optimisation parameters (23 when using twelve control points with correction for arterial delay).

In this work, we propose a non-parametric deconvolution technique using Bézier curves, referred to as Bézier curve deconvolution (BzD). Bézier curves were independently developed by de Casteljau and Bézier in 1959–60 for generation of smooth curves and surfaces for car design [17, 18]. Bézier curves are also frequently used in computer aided graphics design [19]. The main feature of the approach is the use of control polygons where, instead of defining a curve using points on it, points near it are used. Contrary to CPI, where the curve must pass through all control points, a Bézier curve needs only be bounded by the convex hull of its control points. The theory and implementation of Bézier curve deconvolution is shown, and the concept is evaluated using simulated data, including the presence of arterial delay and dispersion and different shapes of the tissue residue function. Proof-of-concept in vivo validation is accomplished by re-evaluation of DSC-MRI data from a healthy volunteer.

## Theory

### Concentration and tracer kinetic theory

The assumption of a linear relationship between the change in transverse relaxation rate $\Delta R{2}^{*}$ and concentration $C(t)$ implies the following relationship [4, 5]:

1 $$C\left(t\right)\propto \Delta R{2}^{*}= -\frac{1}{TE}\mathrm{ln}\left(\frac{S\left(t\right)}{{S}_{0}}\right),$$

where $S(t)$ is the MRI signal at time $t$ and ${S}_{0}$ is the baseline or pre-CA signal.

The AIF is the arterial concentration of contrast agent ${C}_{a}\left(t\right)$ representing the intravascular tracer delivery to the local capillary network. The tissue tracer concentration $C\left(t\right)$ is proportional to the convolution of ${C}_{a}\left(t\right)$ with the tissue impulse response function, i.e., the tissue residue function *R* scaled by the CBF [20]:

2 $$C\left(t\right)=\kappa \cdot CBF\cdot \left({C}_{a}\left(t\right)\otimes R\left(t\right)\right) =\kappa \cdot CBF\cdot {\int }_{0}^{t}{C}_{a}\left(\tau \right)\cdot R\left(t-\tau \right)d\tau ,$$

where $\kappa$ is a constant accounting for the difference in hematocrit levels between capillaries and large vessels and the brain tissue density [21]. Estimation of the product $CBF\cdot R(t)$ via deconvolution provides an estimate of CBF.

MTT can be estimated as the area under the residue function [22]:

3 $$MTT= {\int }_{0}^{\infty }R\left(t\right) dt,$$

and CBV is given by Eq. 4 [4]:

4 $$CBV= \kappa \cdot \frac{{\int }_{0}^{\infty }C\left(t\right) dt}{{\int }_{0}^{\infty }{C}_{a}\left(t\right) dt}.$$

### Bolus delay and dispersion

With a delay of $\delta$ between the measured AIF and the true AIF at the local tissue inlet, the measured AIF is given by ${C}_{a}^{^{\prime}}\left(t\right)={C}_{a}(t-\delta )$, i.e., the AIF shape is retained [15]. Bolus dispersion, on the other hand, modifies the shape and amplitude of the AIF and can be described as a convolution with a vascular transport function (VTF) [10, 11]. When incorporating the effects of bolus delay and dispersion, Eq. 2 can be modified as follows:

5 $$C\left(t\right)=\kappa \cdot CBF\cdot \left({C}_{a}\left(t-\delta \right)\otimes VTF\left(t\right)\otimes R\left(t\right)\right).$$

### Bézier curve deconvolution

Consider the Bernstein polynomials of degree $n$ [23]:

6 $${B}_{i}^{n}\left(\tau \right)=\left(\begin{array}{c}n\\ i\end{array}\right){\tau }^{i}{\left(1-\tau \right)}^{n-i},$$

where $i=0, 1, 2,\dots , n$ and $\tau \in [0, 1]$. A Bézier curve of the ${n}^{th}$ order is a linear combination of these polynomials:

7 $${{\varvec{B}}}_{n}\left(\tau \right)=\sum_{i=0}^{n}{B}_{i}^{n}\left(\tau \right)\cdot {{\varvec{P}}}_{i},$$

where ${{\varvec{P}}}_{i}$ is control point $i$. Expansion for the cubic case ($n=3$) yields

8 $${{\varvec{B}}}_{3}\left(\tau \right)={\left(1-\tau \right)}^{3}{{\varvec{P}}}_{0}+{3\left(1-\tau \right)}^{2}\tau {{\varvec{P}}}_{1}+3\left(1-\tau \right){\tau }^{2}{{\varvec{P}}}_{2}+{\tau }^{3}{{\varvec{P}}}_{3}.$$

**Fig. 1:**
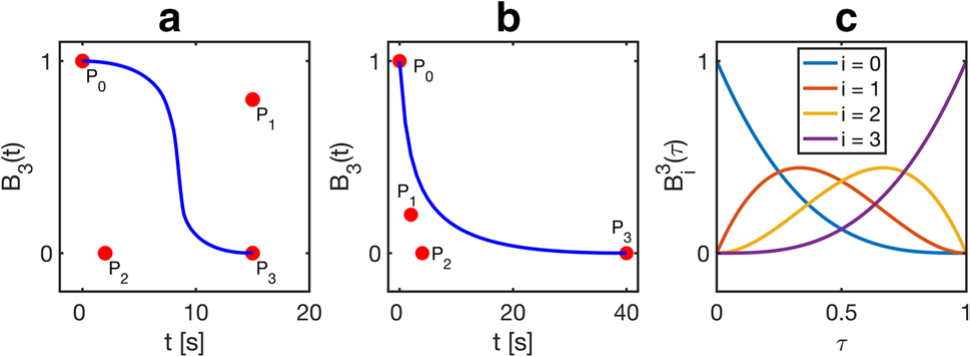
Illustration of the concept of Bézier curves. **a** and **b** show cubic Bézier curves with different control point configurations and **c** shows the corresponding basis polynomials

The control points are effectively weights that determine the influence of each basis polynomial. Equivalently, each point on the Bézier curve is a weighted average of the control points. According to Eq. 8, the Bézier curve invariably begins at ${{\varvec{P}}}_{0}$ and ends at ${{\varvec{P}}}_{n}$. Intermediate control points need not be contained by the curve, but the curve lies wholly within the convex hull of the control points. Figure 1 provides an illustration of this concept using cubic Bézier curves with two different distributions of control points. The clear difference between the two shapes in Fig. 1a and b highlights the versatility of these curves. Figure 1c shows the third-order Bernstein polynomials that form the basis for the cubic Bézier curves. It is important to note that a Bézier curve is fully specified by its control points.

**Fig. 2:**
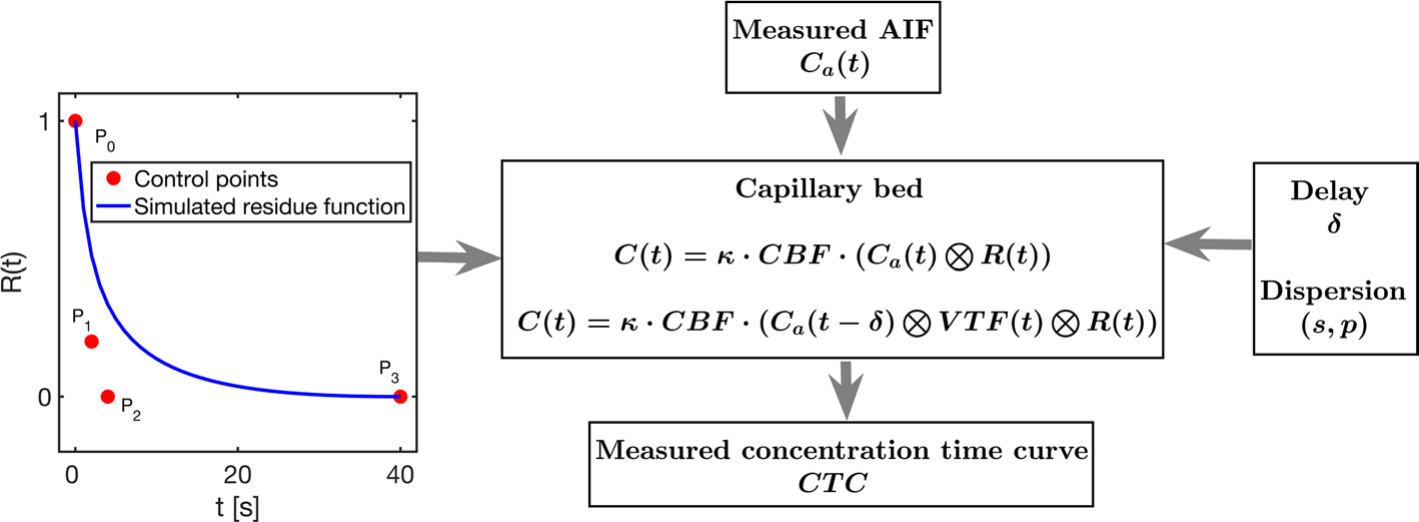
Illustration of Bézier curve deconvolution as a non-linear single-input single-output system. The residue function obtained as a cubic Bézier curve specified by the supplied control points is convolved with the measured AIF (following delay/dispersion) to give a predicted concentration time curve

In the context of deconvolution, the Bézier curve represents the residue function, and the control points constitute parameters to be determined from input data as illustrated by Fig. 2. Parameter fitting is accomplished by comparison of the modeled CTC, as depicted in Fig. 2, with the observed CTC obtained from the measured DSC-MRI signal [11]. BzD assumes a model-based approach to CBF quantification but allows a large distribution of residue function shapes within the constraints specified in the optimisation procedure.

## Materials and methods

### Implementation

Deconvolution methods were implemented in MATLAB using in-house written algorithms. For purposes of comparison, block-circulant SVD (oSVD) deconvolution was implemented according to Wu et al. [15].

BzD was implemented in a Bayesian framework adopting the maximum a posteriori (MAP) approach. The measured signal was converted to concentration using Eq. 1. Following the approach in [9], it was assumed that measurements of concentration over time followed the non-linear observation model:

9 $${Y}_{t}=f\left({C}_{a}, \varphi \right)+{\varepsilon }_{t,}$$

where ${Y}_{t}$ is the observed concentration at time $t$, $f$ represents the right-hand side of Eq. 2, $\varphi$ denotes model parameters and $\varepsilon$ is an observational error belonging to a zero-mean Gaussian distribution. Instead of evaluating the entire posterior distribution, optimisation was performed by seeking the mode. Particularly, the optimal parameters were determined by minimizing the negative logarithm of the factorized posterior [24].

Cubic Bézier curves were used throughout this work. Higher-order curves were associated with elevated computational expense without apparent improvement of outcome (results not shown). The residue function was forced to obey $R\left(0\right)=1$ by fixing the first control point at ${{\varvec{P}}}_{0}=[0, 1]$. In addition, to ensure bounded-input, bounded-output stability, the magnitude of the last control point was fixed at zero (${P}_{3,y}=0$). The x-coordinate remained free. A penalty term was incorporated into the posterior to permit the imposing of constraints. The requirements $0\le {P}_{i,y}\le 1$ and $0\le {P}_{i,x}\le {P}_{n,x}$ were enforced to obtain monotonically decreasing, bijective and positive-valued residue functions. This resulted in 5 optimisation parameters for the generation of the residue function $R(t)$ in the form of a cubic Bézier curve as given by Eq. 8. $R(t)$ was initially evaluated with a 10-ms sampling interval, then resampled to match the arterial input data (i.e., AIF) using cubic spline interpolation. Convolution with the measured AIF and scaling with $\kappa \cdot CBF$ was performed to generate a predicted CTC according to Eq. 2.

#### BzD with delay and dispersion modeling

Delay correction was implemented by shifting the AIF by $\delta$ prior to convolution with the residue function (Eq. 5). For dispersion modeling, the gamma dispersion kernel was chosen as the VTF, as suggested by Mehndiratta et al. [11]:

10 $$VTF\left(t\right)=\frac{{s}^{1+sp}}{\Gamma (1+sp)}\cdot {t}^{sp}\cdot {e}^{-st},$$

where $\Gamma$ denotes the gamma function, $s$ determines the sharpness of the kernel and $p$ is its time-to-peak. In the limit $s\to \infty$ ($p=0$), the kernel approaches the Dirac delta function with no dispersion. Large values of $p$ and small values of $s$ correspond to a higher degree of dispersion [11]. The VTF was convolved with the (shifted) AIF before convolution with the residue function (Eq. 5). Arterial delay ($\delta$) and dispersion ($s,p$) were additional parameters to be determined in the optimisation.

#### Prior knowledge

Bayesian analysis allows the incorporation of prior knowledge into parameter estimation, which can aid in preventing physiologically implausible solutions. Gaussian distributions were chosen for each prior, with mean and standard deviation provided in Table 1. The control points were estimated on a linear scale, with a prior that permitted a wide variety of residue function shapes. A non-informative prior was chosen for CBF, which means setting a standard deviation much larger than the prior mean [11]. The prior for delay was proportional to the difference in time-to-peak between the AIF and measured CTC. Priors for the VTF were adopted from Mehndiratta et al. [11]. The algorithm was initialized at control points that favored a shape intermediate between an exponential and a boxcar residue function, and CBF was set to an arbitrary initial value of 1. Delay and dispersion parameters were initialized at their prior means.

**Table 1:** Prior mean and standard deviations for Bézier curve deconvolution

Parameter	Mean	Standard deviation
$[{{\varvec{P}}}_{1,{\varvec{x}}},\boldsymbol{ }{{\varvec{P}}}_{1,{\varvec{y}}},\boldsymbol{ }{{\varvec{P}}}_{2,{\varvec{x}}},\boldsymbol{ }{{\varvec{P}}}_{2,{\varvec{y}}},\boldsymbol{ }{{\varvec{P}}}_{3,{\varvec{x}}}]$	$[8, 0.5, 2, 0.2, 15]$	$[ 8, 1, 4, 1, 100 ]$
CBF [ml/g/s]	$0.01$	$1{0}^{6}$
Delay ${\varvec{\delta}}$ [s]	$-$	$5$
VTF $({\varvec{s}},{\varvec{p}})$	$[\mathrm{ln}2, \mathrm{ln}2]$	$[2, 2]$

### Simulations

#### AIF, R(t), S(t) and C(t)

A gamma-variate function was used to simulate an AIF, in accordance with previous DSC-MRI simulation studies [7–11]:

11 $$C_{a} (t) = \left\{ \begin{gathered} 0\,\,\,\,\,\,\,\,\,\,\,\,\,\,\,\,\,\,\,\,\,\,\,\,\,\,\,\,\,\,\,\,\,\,\,\,\,\,,\,\,t \le t_{0} \hfill \\ a(t - t_{0} )^{b} e^{{ - (t - t_{0} )/c}} \,\,\,,\,\,t > t_{0} \hfill \\ \end{gathered} \right.$$

where ${t}_{0}=20 \,\mathrm{s}$ is the bolus arrival time, and $a=1, b=3, c=1.5$. The AIF was generated over a time interval of 200 s to prevent signal truncation for the longest MTT simulated in this work (24 s). The sampling interval was $TR=1.24 \,\mathrm{s}$.

**Fig. 3:**
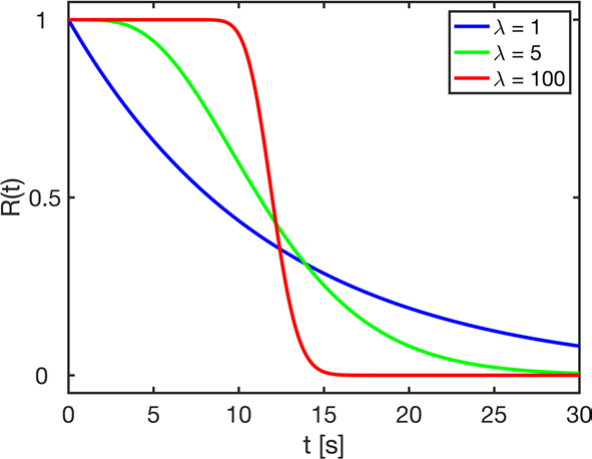
Residue functions belonging to the gamma distribution family, for MTT = 12 s. Values of $\lambda$ of 1, 5 and 100 represent the exponential, sigmoid and boxcar shapes, respectively

Part of the purpose of this work was to evaluate the robustness of BzD to different underlying residue function shapes. Hence, residue functions belonging to the following family of gamma distributions were chosen [8, 25]:

12 $$R\left(t\right)={\int }_{t}^{\infty }\frac{1}{{\beta }^{\lambda }\Gamma (\lambda )}{\tau }^{\lambda -1}{e}^{-\tau /\beta }d\tau ,$$

where $\beta =\frac{MTT}{\lambda }$ with MTT = CBV/CBF and $\lambda$ being a shape parameter. The case $\lambda =1$ corresponds to an exponential residue function, $\lambda$=$100$ was used for the boxcar case and $\lambda =5$ represented a sigmoid shape (intermediate between the exponential and the boxcar) [25]. The three different shapes are depicted in Fig. 3 for MTT = 12 s.

Simulations were performed for a CBV of either 4% (~ 4 ml/100 g) or 2% (~ 2 ml/100 g) representing normal gray and white matter, respectively [7, 10]. From the CTC formed by the convolution of the AIF with the residue function, signal curves were generated using Eq. 1:

13 $$S\left(t\right)={S}_{0}{e}^{-\xi C(t)TE},$$

where ${S}_{0}$ was set to $100$, $TE=29 \,\mathrm{ms}$ and the constant $\xi$ was adjusted to produce a peak signal drop of $40\%$ at a CBF of 60 ml/100 g/min and a CBV of $4\%$, resembling typical values in normal human gray matter [7, 8, 11]. The simulated AIF was also converted to signal ${S}_{a}(t)$ using Eq. 13 but choosing a value of $\xi$ that resulted in a peak signal drop of $60\%$. Zero-mean Rician noise was added to the signal curves, and all simulations were carried out using oSVD and BzD at baseline SNRs of 20 and 100 with $\kappa$ in Eq. 2 set to 1. The oscillation index (OI) for oSVD was fixed at 0.065 for SNR = 100 and 0.035 for SNR = 20 [8]. Examples of the simulated signal data can be found in Supplementary Material 1.

#### No delay, no dispersion

A set of simulations was carried out without any delay or dispersion in the simulated AIF, and with the three residue function shapes shown in Fig. 3. For CBV $=4\%$, CBF was varied from 10 to 70 ml/100 g/min in 10 ml/100 g/min increments, and for CBV $=2\%$, CBF values were simulated in the range $5-35$ ml/100 g/min in 5 ml/100 g/min steps. As a result, the same MTT range ($3.43-24 \,\mathrm{s}$) was obtained for both values of CBV. For each combination of $\lambda$, CBV, CBF and SNR, a total of 1024 CTCs were generated. The remainder of this study considered only the exponential residue function shape; this was motivated by the fact that the exponential exhibits the challenging feature of lacking an initial plateau while still being a standard shape in previous simulation studies.

#### Delay without dispersion

Signal curves were generated after shifting the AIF by $\delta =0, 1, 3$ and $6$ s relative to the simulated residue function, as in [10]. Since these shifts were not integer multiples of $TR$, the AIF was evaluated at a sampling rate of 100 ms, shifted and then resampled to $TR$. This routine was performed for CBV $=4\%$ and CBF in the range 10–70 ml/100 g/min in 10 ml/100 g/min increments. For each combination of $\delta$, CBF and SNR, 100 CTCs were formed. Deconvolution was performed using oSVD and BzD with delay correction.

#### Dispersion without delay

Modeling of dispersion in the absence of delay was achieved by convolving the simulated AIF with a vascular transport function. To avoid using the same VTF as the one in the deconvolution model, an exponential dispersion kernel was selected:

14 $$EDK\left(t\right)=\frac{1}{\theta }{e}^{-\frac{t}{\theta }},$$

where $\theta$ is a time constant for the kernel [11]. Large values of $\theta$ are associated with a higher degree of dispersion and in the limit $\theta \to 0$, the VTF approaches the Dirac delta function with zero dispersion. Low, medium and high dispersion levels were modeled with $\theta =1.5, 3.0$ and $4.5$, respectively [10, 11]. Simulations included CBV $= 4\%$ and CBF $=10, 20, 30, \dots , 70$ ml/100 g/min. For each combination of dispersion level, CBF and SNR, 100 CTCs were generated, with deconvolution being performed using oSVD and BzD with dispersion correction.

#### Dispersion with delay

The simulated AIF was delayed then dispersed as described in the two previous sections. Low, medium and high delay and dispersion were simulated using the combination of parameters $\left[\delta , \theta \right]=[\left\{1 \,\mathrm{s}, 1.5\right\}; \left\{3 \,\mathrm{s}, 3.0\right\}; \{6 \,\mathrm{s}, 4.5\}]$. Simulations were again performed for the same CBV, CBF range and SNR as in the previous two sections, generating 100 CTCs for each combination of these variables. Deconvolution by oSVD and BzD with correction for delay and dispersion was employed.

#### Determination of MTT and rMTT ratios

For each of the three AIF distortions described above (delay, dispersion and delay with dispersion), the quality of MTT estimates was evaluated using two metrics, i.e., the MTT ratio and the rMTT ratio. The MTT ratio was calculated by dividing the estimated MTT by the ground truth value. To mimic a clinical setting, where relative MTT quantification is carried out by normalizing to the MTT in a contralateral region of interest (where the AIF is assumed to be non-distorted), the rMTT ratio was introduced [11]. The rMTT ratio was, thus, defined as the estimated MTT in the presence of a distortion divided by the estimated MTT in the absence of that distortion. For example, let ${MTT}_{0}$ be the estimated MTT at a simulated AIF delay of zero and let ${MTT}_{\delta }$ be the MTT estimate at an AIF delay of $\delta$. The rMTT ratio at the delay of $\delta$ is then given by ${MTT}_{\delta }/{MTT}_{0}$.

#### Accuracy of delay and dispersion parameter estimation

To investigate the performance of the BzD algorithm in the estimation of the parameters describing delay and dispersion, signal curves were generated after dispersing the AIF with the same VTF as used for dispersion correction (gamma dispersion kernel) at an SNR of 100, CBV 4% and CBF 60 ml/100 g/min. Simulations were done at low, medium and high delay and dispersion, with 100 CTCs being created in each case. Different levels of dispersion were modeled by setting the gamma dispersion kernel parameters to $\left[s=2,p=1\right]$, $\left[s=1,p=3\right]$ and $\left[s = 0.5,p=5\right]$ for low, medium and high dispersion, respectively [11]. Analysis was carried out using BzD as described above. Relative errors in the parameters $\delta$ (delay), $p$ (time-to-peak of the VTF) and the sum $(\delta +p)$ were computed as in [11]. Note that both $s$ and $p$ were free in the fitting, but only $p$ was selected for accuracy evaluation. The results of this analysis are presented in Supplementary Materials 4 and 5.

### In vivo data

A proof-of-concept evaluation of BzD was performed through a re-analysis of previously acquired DSC-MRI data from one healthy volunteer [26]. Image acquisition parameters for the DSC-MRI experiment are given in [26]. In brief, the healthy volunteer was scanned at 3 T and a global AIF was sampled from the middle cerebral artery branches in the Sylvian fissure region. A prebolus administration of a low dose of contrast agent (CA) was used to retrieve a venous output function (VOF) from the sagittal sinus prior to the main DSC-MRI experiment. After correcting the VOF area-under-curve (AUC) for the lower CA dose, a subject-specific correction factor for partial volume effects was obtained as the ratio AUC(AIF)/AUC(VOF). The same correction factor as was used by Knutsson et al. [26] was applied in the present study. The OI for oSVD was set to 0.095 [8] and $\kappa$ in Eq. 2 was set to 0.705 ${\mathrm{cm}}^{3}/\mathrm{g}$ [21]. Whole-brain CBF and MTT means were computed after exclusion of pixels with values above 2.5 times the mean over the entire volume as these values were assumed to originate from large vessels.

## Results

### Simulations

#### No delay, no dispersion

**Fig. 4:**
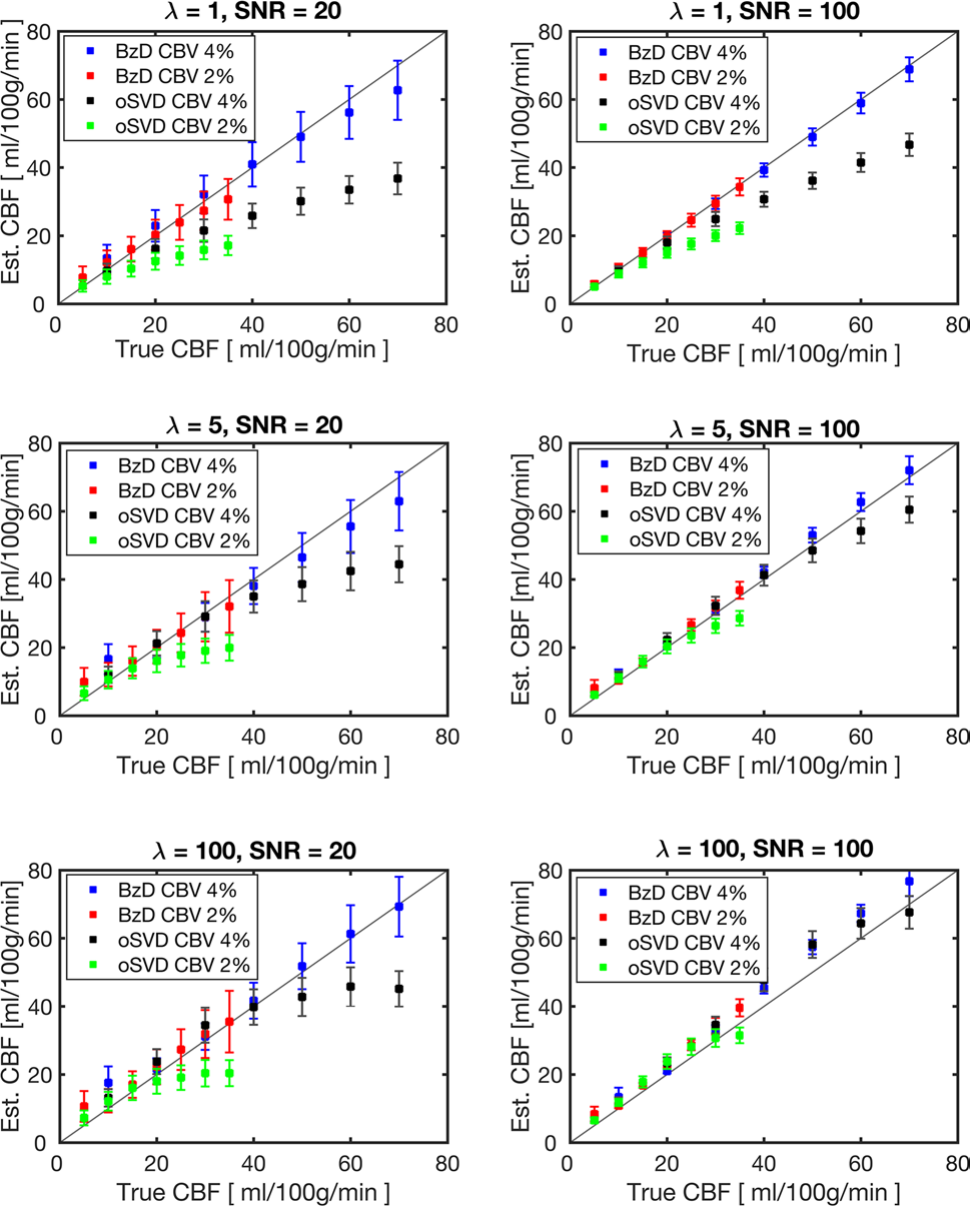
Estimated CBF plotted against true CBF for CBV = 4% and 2%, SNR = 20 and 100 and $\lambda =1, 5$ and 100, modeling the exponential, sigmoid and boxcar residue functions shapes, respectively. Results are shown for Bézier curve deconvolution and oSVD

Figure 4 summarizes estimated CBF against true CBF for the three different underlying residue function shapes. Each point in the plots represents the mean and standard deviation from 1024 noise realizations. BzD estimates were consistently closer to the true CBF than the corresponding oSVD estimates for the exponential residue function ($\lambda =1$). For $\lambda =1$ and $\lambda =5$, BzD tended to slightly underestimate high CBF values when SNR = 20, for both CBV levels. The BzD method exhibited excellent agreement with true CBF values at SNR = 100. Underestimation of high CBF values by BzD was not evident in the boxcar ($\lambda =100$) case, with even some degree of overestimation being observed at SNR = 100. While lower CBF values were generally correctly estimated by both methods, oSVD consistently failed to reproduce high CBF components (i.e., short MTT). Generally, oSVD estimates showed lower variation about their means at low SNR than the corresponding estimates with BzD. Supplementary Material 2 shows the corresponding CBF estimates, for CBV = 4% and SNR = 20, in the presence of delay and dispersion for an underlying exponential residue function.

To obtain a CBF-independent metric to conveniently compare BzD and oSVD, the CBF ratio was computed over the simulated CBF range for a given $\lambda$, CBV and SNR. These results are presented in Table 2, indicating that BzD outperformed oSVD given an exponential residue function and, generally, also given a sigmoid shape. For the boxcar residue function at SNR = 20, oSVD mean ratios were closer to unity than those of BzD. The performances of the two methods for boxcar shape and SNR = 100 were comparable.

**Table 2:** Mean CBF ratios obtained with BzD and oSVD for three different residue function shapes, SNR = 20 and 100

	SNR = 20, CBV = 4%			SNR = 100, CBV = 4%		
	$\lambda =1$	$\lambda =5$	$\lambda =100$	$\lambda =1$	$\lambda =5$	$\lambda =100$
BzD	$1.01\pm 0.12$	$1.05\pm 0.27$	$1.13\pm 0.27$	$1.02\pm 0.04$	$1.06\pm 0.05$	$1.14\pm 0.09$
oSVD	$0.69\pm 0.16$	$0.89\pm 0.20$	$0.99\pm 0.24$	$0.80\pm 0.12$	$1.02\pm 0.11$	$1.13\pm 0.09$

	SNR = 20, CBV = 2%			SNR = 100, CBV = 2%		
BzD	$1.06\pm 0.19$	$1.16\pm 0.38$	$1.25\pm 0.39$	$1.03\pm 0.08$	$1.14\pm 0.22$	$1.21\pm 0.21$
oSVD	$0.68\pm 0.19$	$0.86\pm 0.26$	$0.95\pm 0.31$	$0.78\pm 0.13$	$1.01\pm 0.14$	$1.13\pm 0.13$

The results show the mean and standard deviation of the CBF ratios for the ranges 10 – 70 ml/100 g/min (CBV = 4%) and 5 – 35 ml/100 g/min (CBV = 2%)

**Fig. 5:**
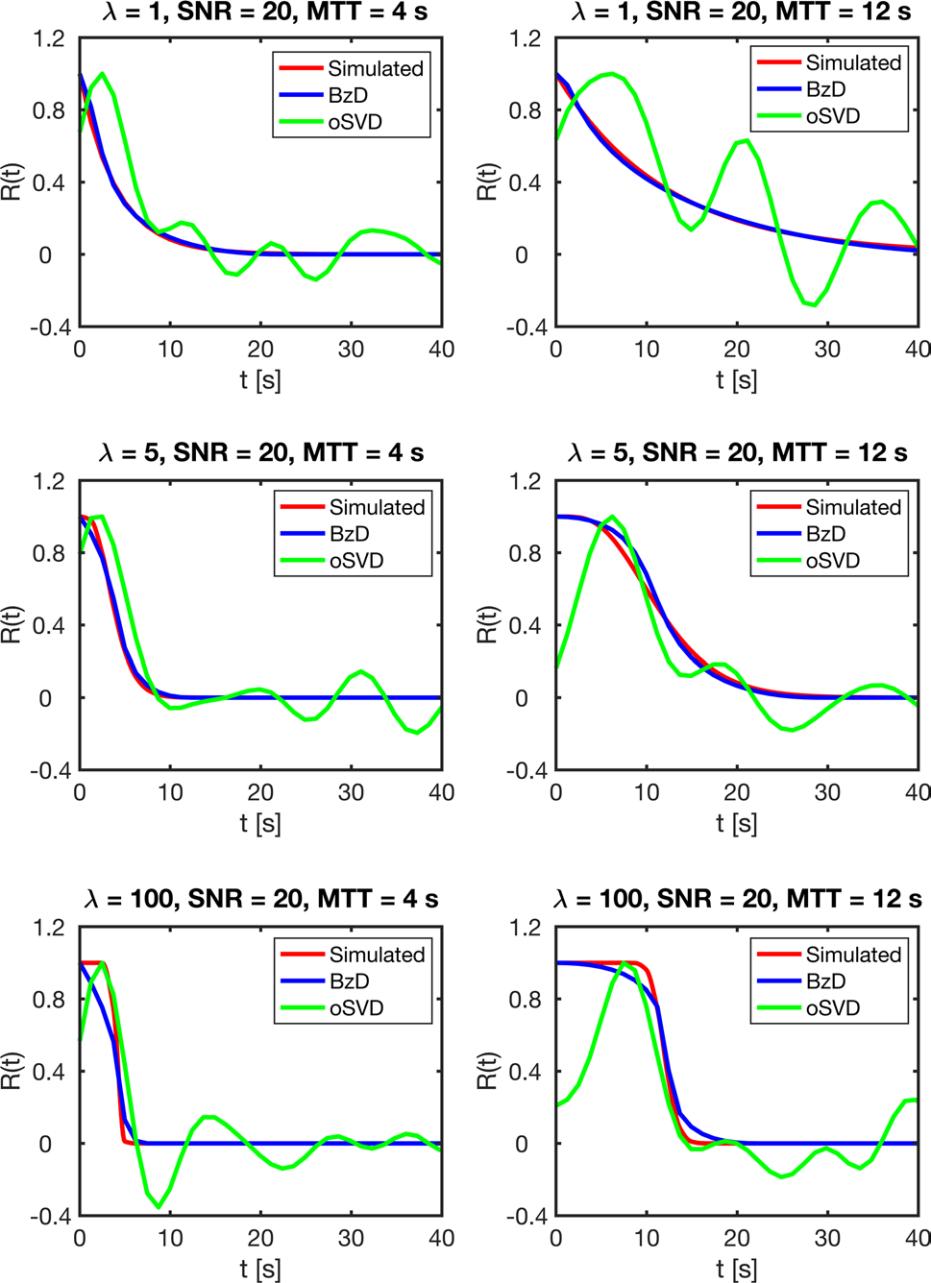
Typical residue functions obtained in simulation with BzD and oSVD, using an SNR of 20, and MTTs of 4 s and 12 s, for three different underlying residue function shapes

**Fig. 6:**
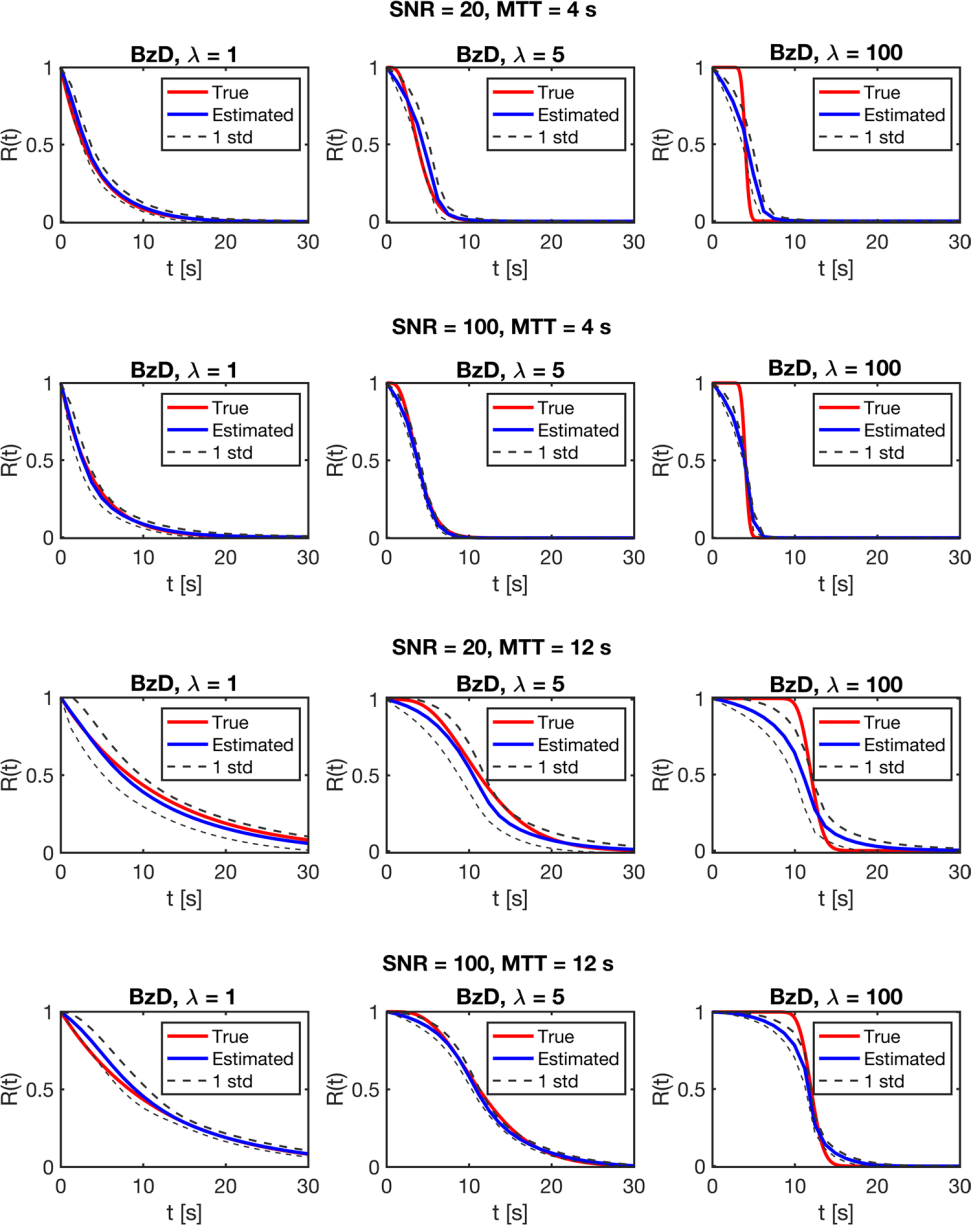
Mean residue function fit estimated with BzD, for MTT = 4 s (CBF 60 ml/100 g/min for a CBV of 4%) and 12 s (CBF of 20 ml/100 g/min for a CBV of 4%), for SNR = 20 and 100. The dotted lines represent one standard deviation

Examples of residue functions obtained with BzD and oSVD are presented in Fig. 5, for SNR = 20, MTT = 4 s (CBV = 4%, CBF = 60 ml/100 g/min) and MTT = 12 s (CBV = 4%, CBF = 20 ml/100 g/min). BzD produced smooth, monotonically decreasing, non-negative residue functions that were free of the oscillatory behavior that characterized the corresponding oSVD solutions. Moreover, BzD was in all cases in superior agreement with the true underlying residue function compared to oSVD. This latter finding is quantified more systematically in Table 3, using the root-mean-square error (RMSE) as a measure of accuracy of residue function estimation. According to Table 3, BzD provided more accurate estimates of residue function shape than oSVD in all simulated scenarios. The accuracy of residue function shape estimation with BzD declined as the underlying shape deviated from an exponential (that is, as $\lambda$ increased); this result is coherent with the trend observed for the CBF estimation. Furthermore, for a given simulated residue function shape, the accuracy was invariably higher for higher CBV (Table 3) and for shorter MTT (Fig. 6).

**Table 3:** Root-mean-square errors in residue function shape estimation, showing the mean and standard deviation over the CBF range 10–70 ml/100 g/min (CBV = 4%) and 5–35 ml/100 g/min (CBV = 2%)

	SNR = 20, CBV = 4%			SNR = 100, CBV = 4%		
	$\lambda =1$	$\lambda =5$	$\lambda =100$	$\lambda =1$	$\lambda =5$	$\lambda =100$
BzD	$0.03\pm 0.02$	$0.04\pm 0.05$	$0.06\pm 0.06$	$0.02\pm 0.01$	$0.02\pm 0.02$	$0.04\pm 0.04$
oSVD	$0.13\pm 0.03$	$0.12\pm 0.04$	$0.13\pm 0.04$	$0.08\pm 0.02$	$0.08\pm 0.02$	$0.08\pm 0.02$

	SNR = 20, CBV = 2%			SNR = 100, CBV = 2%		
BzD	$0.04\pm 0.03$	$0.06\pm 0.06$	$0.08\pm 0.07$	$0.02\pm 0.02$	$0.03\pm 0.05$	$0.06\pm 0.06$
oSVD	$0.19\pm 0.05$	$0.18\pm 0.06$	$0.19\pm 0.06$	$0.10\pm 0.02$	$0.10\pm 0.03$	$0.10\pm 0.03$

The results are presented for three different underlying residue function shapes, SNR = 20 and SNR = 100 with both oSVD and BzD

A visual appreciation of the results in Table 3 is given in Fig. 6, which shows the mean residue function fit obtained with BzD, plus/minus one standard deviation. Results are shown for all three simulated shapes, MTT = 4 s (CBV 4%, CBF 60 ml/100 g/min) and MTT = 12 s (CBV 4%, CBF 20 ml/100 g/min), both for SNR = 20 and 100.

#### Delay without dispersion

**Fig. 7:**
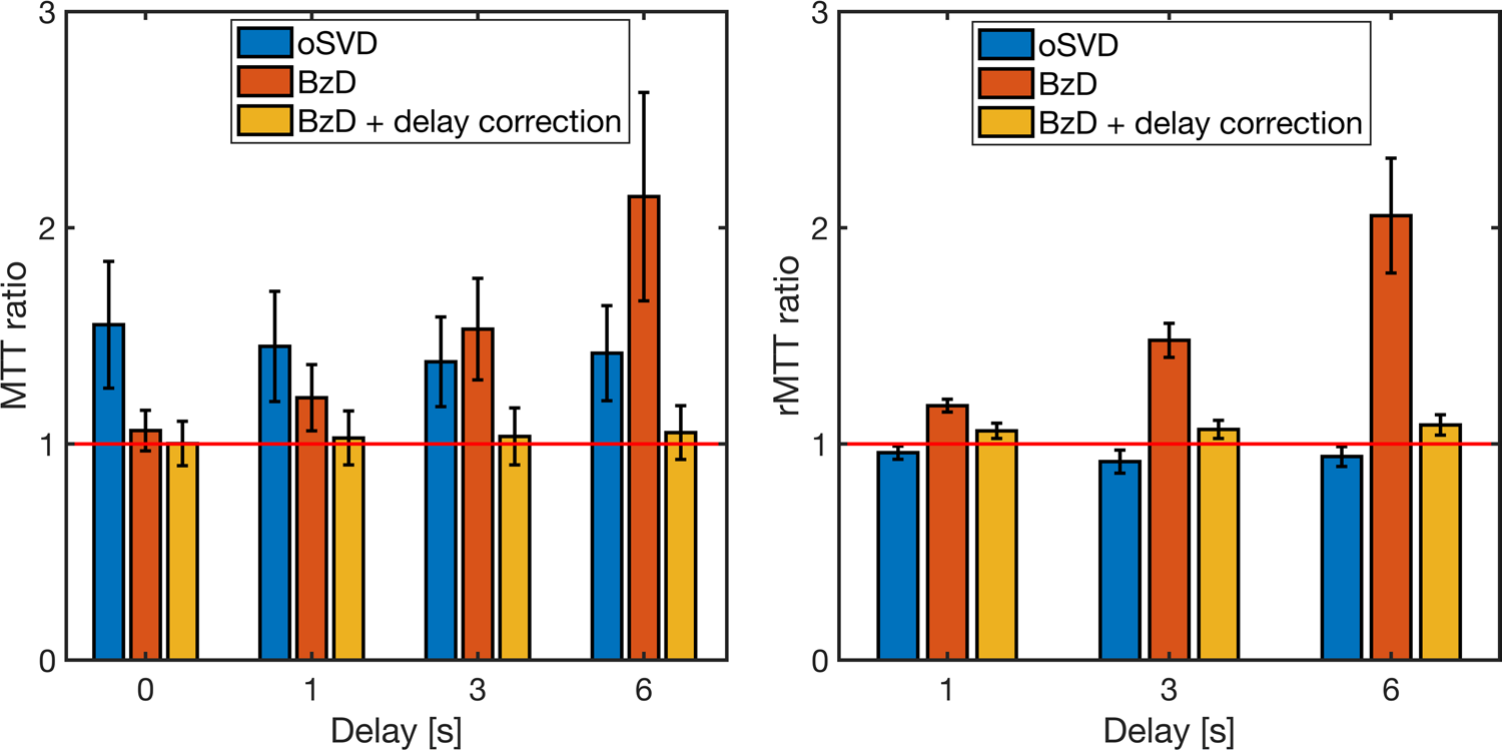
MTT ratio (left) and rMTT ratio (right) for oSVD, BzD and BzD with delay correction. Each bar represents the mean and standard deviation over MTT estimates for underlying CBF values in the range [10–70] ml/100 g/min, a CBV of 4% and SNR 20

The accuracy of MTT estimation with BzD, BzD with delay correction and oSVD is summarized in Fig. 7. MTT and rMTT ratios were averaged over the range 3.43–24 s corresponding to CBF in the range 10–70 ml/100 g/min for a CBV of 4% and SNR = 20. The oSVD method overestimated MTT but demonstrated consistency over all simulated shifts. BzD without delay correction overestimated MTT, with its performance diminishing as the delay increased. Excellent agreement with true MTT values was achieved with BzD including delay correction. The rMTT ratios for oSVD and BzD with delay correction were close to unity across all simulated delays, consistent with the observed trend in MTT ratios. A slight underestimation was seen in the rMTT ratios for oSVD. BzD without delay correction displayed a sensitivity to delay, producing successively higher rMTT ratios as the delay increased.

#### Dispersion without delay

**Fig. 8:**
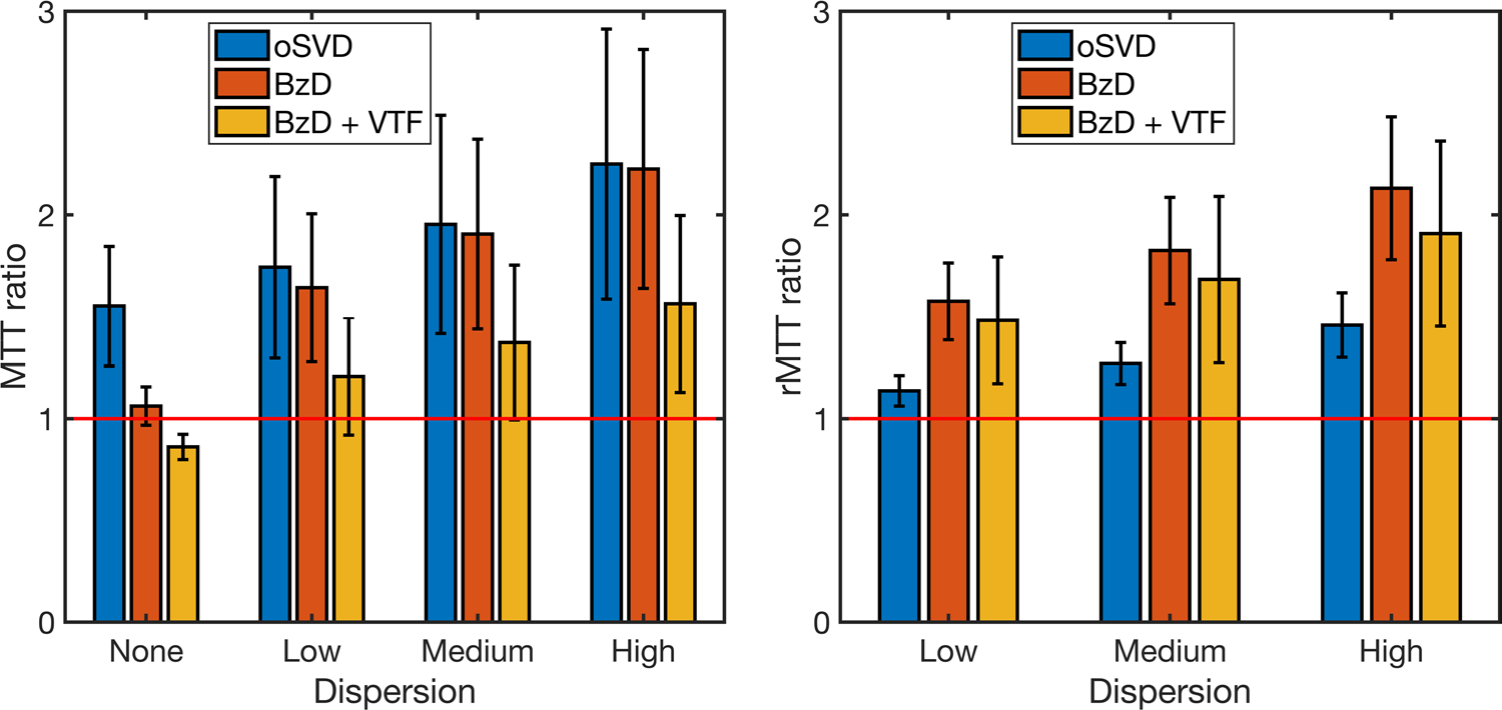
MTT ratio (left) and rMTT ratio (right) for oSVD, BzD and BzD with dispersion correction. The bars show the mean and standard deviation over MTT estimates for underlying CBF values in the range [10–70] ml/100 g/min, a CBV of 4% and SNR 20

The corresponding results for the case of dispersion without delay are presented in Fig. 8. MTT overestimation is apparent with oSVD, and the method exhibited a gradual decline in performance from low to high dispersion. BzD without dispersion correction displayed marked sensitivity to dispersion, showing a performance matching that of oSVD at all levels of the effect. Incorporation of a VTF into BzD drastically improved MTT estimation for low, medium and high dispersion, but led, on the other hand, to underestimation of MTT in the absence of this effect. The rMTT ratios for both oSVD and BzD with dispersion correction drifted further from unity with increasing level of dispersion. The performance of oSVD for relative MTT quantification (as captured by the rMTT ratios) was higher than that of BzD with dispersion correction, while BzD without correction for dispersion returned the worst results.

#### Delay and dispersion

**Fig. 9:**
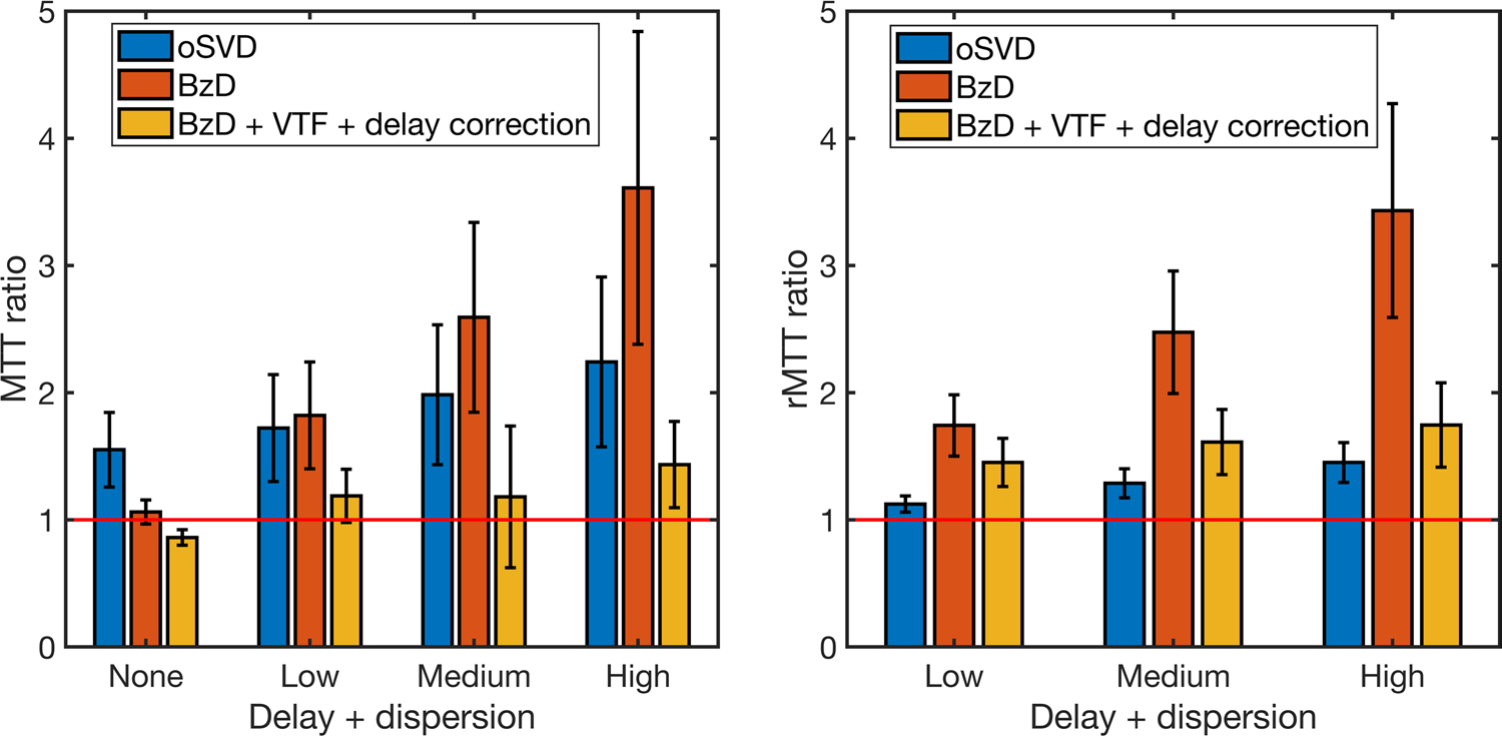
MTT ratio (left) and rMTT ratio (right) for oSVD, BzD and BzD with correction for both delay and dispersion. The results show the mean and standard deviation over MTT estimates for underlying CBF values in the range [10–70] ml/100 g/min, a CBV of 4% and SNR 20

Shown in Fig. 9 are the MTT and rMTT ratios for the case of delay coupled with dispersion. A rather similar trend to the one observed in Fig. 8 (dispersion without delay) is evident. BzD without correction for delay or dispersion exhibited lower performance when delay was combined with dispersion than with dispersion alone. When equipped with correction for both delay and dispersion, BzD appeared to outperform oSVD for absolute estimation and parallel oSVD for relative estimation. Finally, note that results similar to those shown in Figs. 7, 8, and 9 can be found in Supplementary Material 3 for an SNR of 100.

#### Residue functions with delay and dispersion

**Fig. 10:**
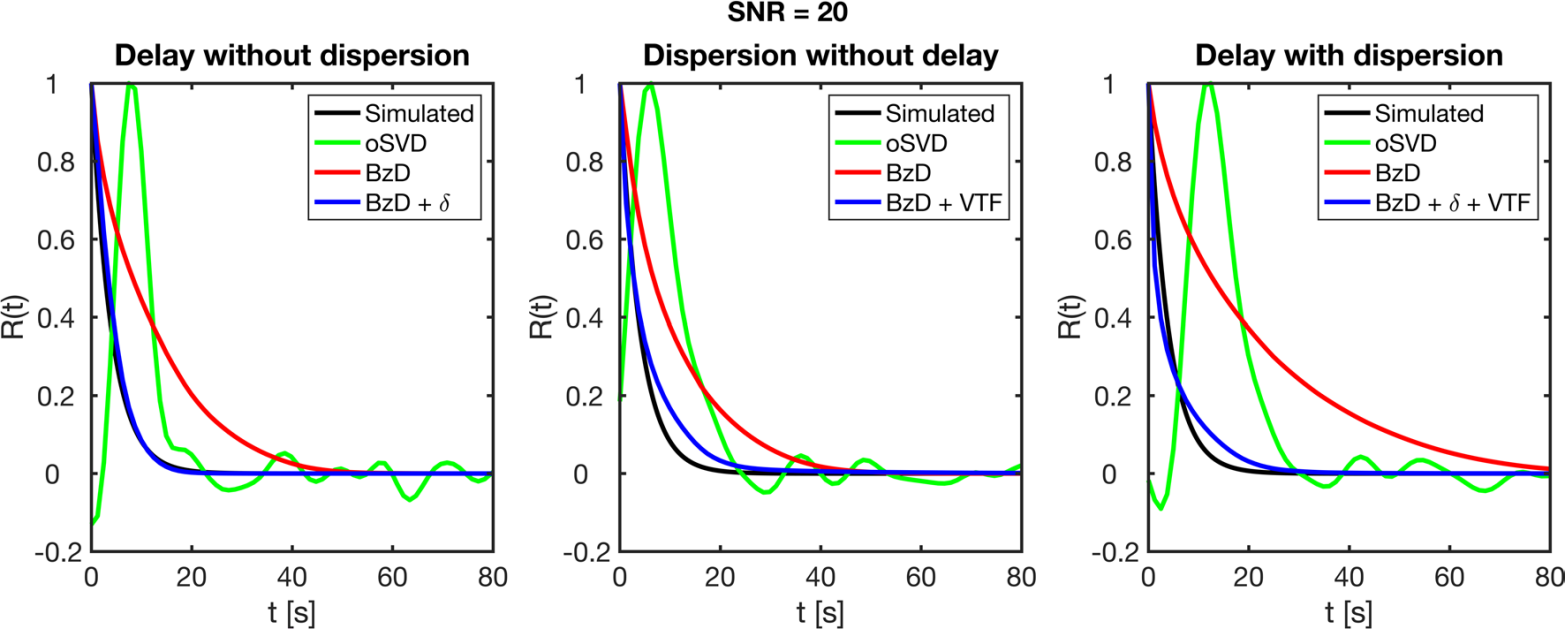
Mean residue function fit produced with oSVD, BzD and BzD with correction for delay and/or dispersion. The plots were generated in the presence of high delay without dispersion (left), high dispersion without delay (middle) and high delay combined with high dispersion (right), at CBV = 4%, CBF = 60 ml/100 g/min and SNR = 20

Evaluation of the ability to reproduce the residue function shape in the presence of delay and dispersion is summarized in Fig. 10. The plots show mean estimated residue functions for BzD (with and without correction) and oSVD, for the cases of high delay without dispersion, high dispersion without delay and high delay and dispersion. In each case, CBV was 4%, CBF 60 ml/100 g/min (typical gray matter) and SNR 20. The estimated residue function in the presence of delay and/or dispersion was characterized by a slower decay relative to the simulated shape. BzD with correction for delay and/or dispersion brought the resulting residue function into close agreement with the underlying true shape.

### In vivo data

**Fig. 11:**
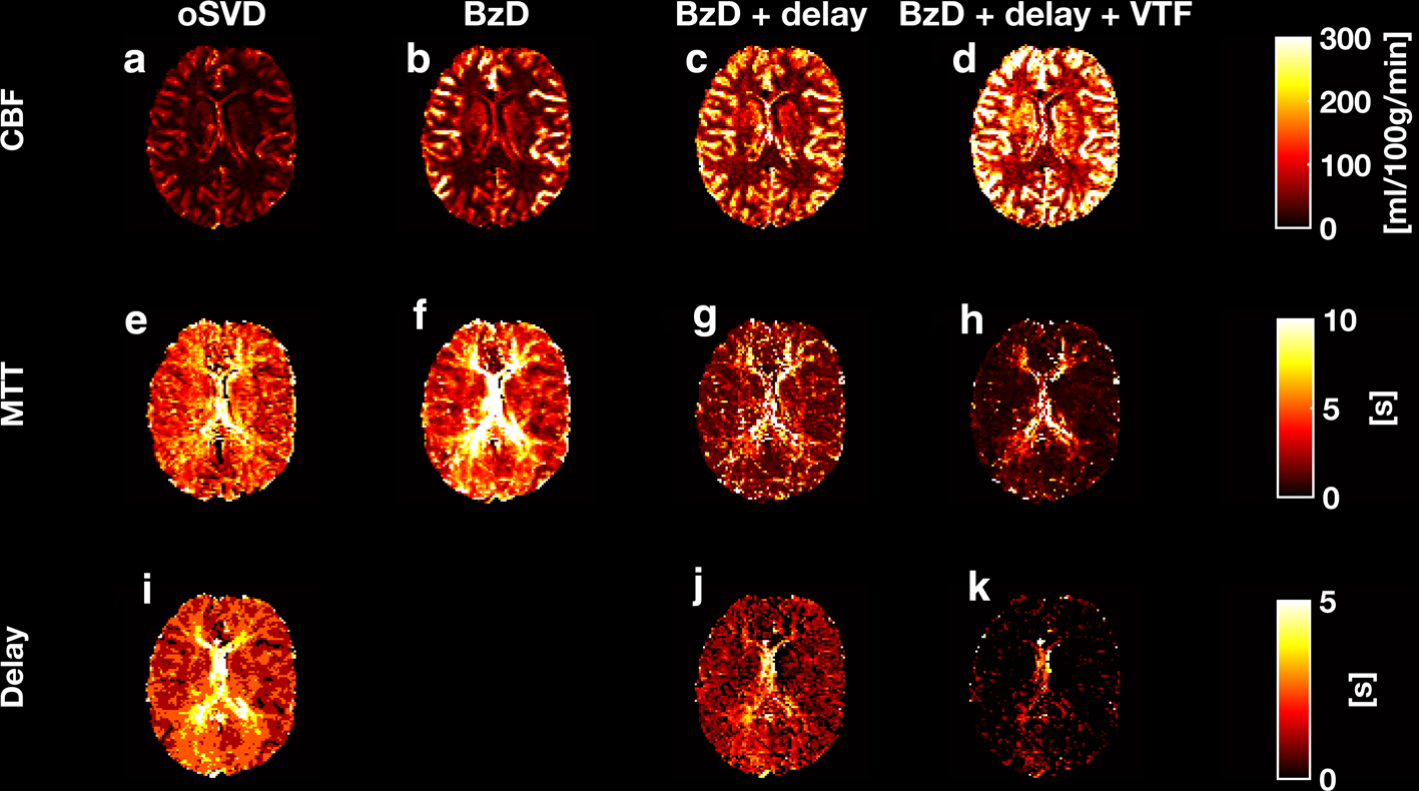
CBF, MTT and delay maps from the analysis of in vivo DSC-MRI data from a healthy volunteer using oSVD, BzD, BzD with delay correction and BzD with correction for both delay and dispersion

Parametric maps from the analysis of clinical data with oSVD, BzD, BzD with delay correction and BzD with correction for delay and dispersion are shown in Fig. 11. BzD in any of the presented variants produced higher flow estimates than oSVD. The whole-brain CBF means for the four methods were: oSVD (42 $\pm$ 27), BzD (58 $\pm$ 41), BzD + delay (100 $\pm$ 70) and BzD + delay + VTF (150 $\pm$ 100) ml/100 g/min. Delay correction with BzD led to an increase in the calculated CBF and the incorporation of a VTF achieved the same effect.

The MTT maps also showed variation among the four presented scenarios, with highest values being observed with oSVD (4.9 $\pm$ 2.8) and BzD (5.3 $\pm$ 5.8) s. MTT values for BzD methods decreased in the following order: BzD, BzD with delay correction (3.2 $\pm$ 4.6) s, BzD with delay and dispersion correction (1.8 $\pm$ 4.2) s. There was some discrepancy between oSVD and BzD in the delay estimation, with oSVD producing notably higher delay values than both variants of BzD shown. Interestingly, BzD with delay correction estimated higher values of delay than the same method incorporating a VTF.

**Fig. 12:**
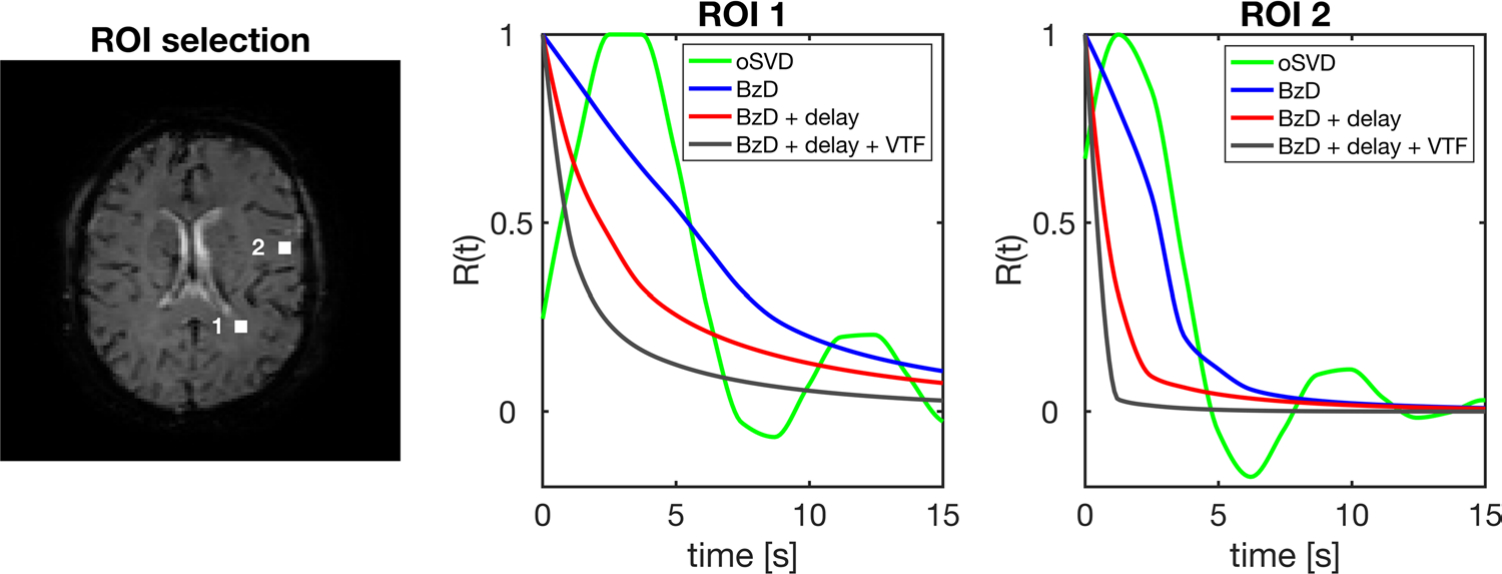
In vivo residue function shapes obtained with oSVD, BzD and BzD including correction for dispersion and/or delay. The plots reflect mean solutions over the 4 × 4 pixel ROIs

Residue function shape estimation in vivo is shown in Fig. 12, for the four methods of analysis displayed in Fig. 11. The residue functions plotted are means over 4 × 4 pixels in two different ROIs. Solutions from oSVD are oscillatory, while BzD produces smooth and monotonically decaying residue functions. For both ROIs, correction for either delay or delay and dispersion results in the same effect. The residue function plots are strongly reminiscent of the results presented in Fig. 10.

## Discussion

Retrieval of the tissue impulse response function from the measured concentration curve and the AIF has been a persistent challenge in DSC-MRI, despite the introduction of a number of relevant mathematical methods. Recent studies include stable spline deconvolution [27], deconvolution with dispersion-compliant bases [28] assessment of the robustness of spatio-temporal deconvolution algorithms [29] as well as the introduction of machine learning approaches to DSC-MRI perfusion imaging [30]. In the present study, a non-parametric deconvolution method using Bézier curves for perfusion quantification in DSC-MRI was presented and evaluated.

In the absence of delay and dispersion, Bézier curve deconvolution showed obvious robustness to different underlying residue function shapes. The method produced accurate perfusion estimates and outperformed oSVD, except in the most extreme case (boxcar residue function at an SNR of 20). CBF estimates from BzD were accompanied by smooth, physiologically plausible tissue residue functions that obeyed the fundamental requirements of non-negative values and monotonical decrease, and they were, thus, free of the oscillations typically seen for SVD-based deconvolution methods. It is not surprising that BzD exhibited lower performance with a boxcar residue function than with the sigmoid or exponential shapes. Complete representation of such a shape (exhibiting a non-smooth behavior) using Bézier curves requires a relatively complex configuration of control points, rendering it an unlikely solution when using cubic curves. Bézier curves are inherently smooth, and thus, they favor smooth functions such as the exponential/sigmoid. It is in principle possible to bring the calculated residue function into good agreement with the boxcar shape using higher-order curves (more control points), but as highlighted earlier, this was not within the scope of the current implementation. Implementations with fourth order curves (results not shown) significantly increased computation times, and higher-order curves also had adverse effects on the accuracy of CBF estimation when combined with correction for delay and/or dispersion with an exponential underlying residue function. Incorporation of delay and dispersion correction was judged to be crucial, while accurate representation of a non-smooth residue curve had lower priority, given that non-smooth behavior is not physiologically plausible and functions resembling the exponential/sigmoid shapes are a more reasonable representation of true physiology. However, the estimates to the boxcar residue function obtained with BzD in this study appear, from visual inspection, to show closer agreement with the true shape than those obtained with the CPI method by Mehndiratta et al. [7].

BzD including a correction for delay was able to correctly reproduce MTT values in the range 3.4–24 s, for shifts of up to 6 s. Accurate estimates were also obtained by BzD with corrections for dispersion as well as for delay with dispersion, except for some degree of underestimation for zero and low levels of each effect. Zero dispersion is only achieved when the gamma dispersion kernel approaches a delta function (i.e., the sharpness parameter $s$ in the VTF must approach infinity for a time-to-peak of zero). Convergence to these values of the VTF parameters did not occur, giving rise to the observed MTT underestimation. Relative quantification results showed comparable performance between oSVD and BzD with delay and/or dispersion correction. Furthermore, oSVD overestimated MTT at all simulated shifts and dispersion levels but demonstrated a consistency that emanates from its inherent insensitivity to bolus delay. Hence, in the presence of delay and dispersion, for absolute perfusion quantification, oSVD parallels BzD when neither delay nor dispersion is corrected for. Under the same circumstances, BzD with a correction largely outperforms oSVD. For relative quantification, BzD with correction and oSVD are equally suitable, both surpassing BzD without delay or dispersion correction.

BzD without corrections provided smooth residue functions also in the presence of delay and dispersion, but both effects being signified by a spread-out solution (slower decay of the estimated residue function). Correction for delay and dispersion restored the estimated residue function to good agreement with the true shape. This result illustrates that accurate perfusion estimation with BzD, in the presence of delay and dispersion, is accompanied by physiologically plausible residue functions.

CBF maps attained from in vivo data were in general correspondence with the trends seen in the simulations, with the highest CBF values being estimated with BzD accounting for both delay and dispersion effects. Whole-brain mean CBF obtained with oSVD and BzD were in harmony with values estimated in a previous analysis of the same data [26] and with values measured with positron emission tomography [31]. However, BzD produced higher blood flow estimates with larger variability than oSVD, a result which mirrors the findings presented in Table 2. The higher spread in CBF estimates with BzD is believed to originate from low CBV regions, and we note that a more comprehensive comparison between oSVD and BzD might include a larger range of CBV values. In future work, application of both oSVD and BzD in stroke or cancer patients would also provide a more realistic comparison. BzD with delay correction, and particularly BzD with correction for both delay and dispersion, tended to overestimate CBF. This is to be expected, and it implies that low levels of delay and dispersion prevailed (cf. Figures 7, 8, 9 and Supplementary Material 5), which seems reasonable considering that the DSC-MRI data were acquired from a healthy volunteer. Very low levels of dispersion might, as observed in the simulations (cf. Figure 9), have led to an overestimation of CBF (i.e., an underestimation of MTT) when correcting for both delay and dispersion. MTT values followed the inverse of the pattern observed in CBF maps, in agreement with the central volume theorem. The result is also reasonable when viewing MTT as the area under the residue function; simulations established that both delay and dispersion correction led to a faster decay of the residue function, which is associated with a shorter MTT.

The observation that oSVD produced higher delay estimates than BzD in vivo is likely a consequence of the oscillatory nature of the oSVD residue functions coupled with the current definition of delay as the time point at which the residue function attains its maximum value. BzD with delay correction and BzD with delay and dispersion correction exhibited a notable discrepancy in delay estimation in vivo. This finding constitutes a practical example of the discussion pertaining to the results in Table S1 (Supplementary Material 4), namely that delay and dispersion are corrected in combination rather than as individual effects.

BzD also gave smooth residue functions in vivo and the effects of delay and dispersion correction reflected those observed in the simulations. An additional highlight of the in vivo results is the shape variability between the two sampled regions. It can be inferred that BzD is able to reflect in vivo residue functions with both fast and slow decay rates.

In conclusion, Bézier curve deconvolution bears substantial promise for perfusion quantification in DSC-MRI. It is a robust, non-parametric deconvolution technique that generally provides more accurate perfusion estimates than oSVD, along with realistically smooth residue functions. The power of the algorithm lies in its employment of the intrinsically smooth Bézier curves, the small number of parameters that require optimisation, and the ability to incorporate appropriate prior knowledge into the deconvolution process. We envision Bézier curve deconvolution to be of clinical value in the investigation of patients with cerebrovascular diseases, where dispersion effects are known to corrupt oSVD-based estimates of perfusion-related parameters [7, 10]. For instance, more reliable perfusion estimates obtained with the proposed approach can enable improved delineation of the salvageable ischemic penumbra in stroke patients. Furthermore, the availability of physiologically plausible residue functions opens an important avenue of research into the diagnostic potential of the residue function shape, for example, for assessment of cerebral oxygen extraction [9].

## Supplementary Information

Below is the link to the electronic supplementary material.

Supplementary file1 (TIF 10654 KB) Simulated DSC-MRI data examples for MTT = 4s (top) and MTT = 24 s (bottom). The images (labeled “Image at 27 s”) show the simulated typical gray matter and white matter pixels. The data were generated at SNR = 20

Supplementary file2 (TIF 7601 KB) CBF estimates, for CBV = 4% and SNR = 20, obtained with oSVD and BzD in the presence of delay and dispersion using an exponential residue function. These results are to be compared to Fig. 4 in the main text, which shows corresponding estimates in the absence of delay or dispersion

Supplementary file3 (TIF 31707 KB) MTT and rMTT ratios from the study of delay and dispersion at SNR = 100. Similar results are shown for an SNR of 20 in Figs 7–9 in the main text

Supplementary file4 (PDF) Accuracy of delay and dispersion parameter estimation

Supplementary file5 (TIF 23053 KB) MTT estimates from BzD and oSVD obtained from the study of delay correction in the presence of dispersion without delay. Notice how BzD with delay correction improves MTT estimates even though delay is absent

## Data Availability

Source code and simulated data have been shared in a public repository: https://github.com/arthur-chakwizira/BezierCurveDeconvolution

## References

[1] Wu O, Koroshetz WJ, Østergaard L, Buonanno FS, Copen WA, Gonzalez RG, Rordorf G, Rosen BR, Schwamm LH, Weisskoff RM, Sorensen AG (2001). Predicting tissue outcome in acute human cerebral ischemia using combined diffusion- and perfusion-weighted MR imaging. Stroke.

[2] Hirano T (2014). Searching for salvageable brain: The detection of ischemic penumbra using various imaging modalities?. J Stroke Cerebrovasc Dis.

[3] Waqar M, Lewis D, Agushi E, Gittins M, Jackson A, Coope D (2021). Cerebral and tumoral blood flow in adult gliomas: a systematic review of results from magnetic resonance imaging. Br J Radiol.

[4] Rosen BR, Belliveau JW, Buchbinder BR, McKinstry RC, Porkka LM, Kennedy DN, Neuder MS, Fisel CR, Aronen HJ, Kwong KK, Weisskoff RM, Cohen MS, Brady TJ (1991). Contrast agents and cerebral hemodynamics. Magn Reson Med.

[5] Østergaard L, Weisskoff RM, Chesler DA, Gyldensted C, Rosen BR (1996). High resolution measurement of cerebral blood flow using intravascular tracer bolus passages. Part I: Mathematical approach and statistical analysis. Magn Reson Med.

[6] Zierler KL (1962). Theoretical basis of indicator-dilution methods for measuring flow and volume. Circ Res.

[7] Mehndiratta A, MacIntosh BJ, Crane DE, Payne SJ, Chappell MA (2013). A control point interpolation method for the non-parametric quantification of cerebral haemodynamics from dynamic susceptibility contrast MRI. Neuroimage.

[8] Mouridsen K, Friston K, Hjort N, Gyldensted L, Østergaard L, Kiebel S (2006). Bayesian estimation of cerebral perfusion using a physiological model of microvasculature. Neuroimage.

[9] Mouridsen K, Hansen MB, Østergaard L, Jespersen SN (2014). Reliable estimation of capillary transit time distributions using DSC-MRI. J Cereb Blood Flow Metab.

[10] Calamante F, Gadian DG, Connelly A (2000). Delay and dispersion effects in dynamic susceptibility contrast MRI: simulations using singular value decomposition. Magn Reson Med.

[11] Mehndiratta A, Calamante F, MacIntosh BJ, Crane DE, Payne SJ, Chappell MA (2014). Modeling and correction of bolus dispersion effects in dynamic susceptibility contrast MRI. Magn Reson Med.

[12] Calamante F, Gadian D, Connelly A (2002). Quantification of perfusion using bolus tracking magnetic resonance imaging in stroke. Stroke.

[13] Zaharchuk G, Bammer R, Straka M, Newbould RD, Rosenberg J, Olivot J-M, Mlynash M, Lansberg MG, Schwartz NE, Marks MM, Albers GW, Moseley ME (2009). Improving dynamic susceptibility contrast MRI measurement of quantitative cerebral blood flow using corrections for partial volume and nonlinear contrast relaxivity: a Xenon CT comparative study. J Magn Reson Imaging.

[14] Gobbel GT, Fike JR (1994). A deconvolution method for evaluating indicator-dilution curves. Phys Med Biol.

[15] Wu O, Østergaard L, Weisskoff RM, Benner T, Rosen BR, Sorensen AG (2003). Tracer arrival timing-insensitive technique for estimating flow in MR perfusion-weighted imaging using singular value decomposition with a block-circulant deconvolution matrix. Magn Reson Med.

[16] Calamante F, Gadian DG, Connelly A (2003). Quantification of bolus-tracking MRI: improved characterization of the tissue residue function using Tikhonov regularization. Magn Reson Med.

[17] Fitter HN, Pandey AB, Patel DD, Mistry JM (2014). A review on approaches for handling Bezier curves in CAD for manufacturing. Procedia Eng.

[18] Baydas S, Karakas B (2019). Defining a curve as a Bezier curve. J Taibah Univ Sci.

[19] Farin G, Bézier P, Farin G (2002). How a simple system was born. Curves and surfaces for CAGD.

[20] Zierler KL (1965). Equations for measuring blood flow by external monitoring of radioisotopes. Circ Res.

[21] Rempp KA, Brix G, Wenz F, Becker CR, Gückel F, Lorenz WJ (1994). Quantification of regional cerebral blood flow and volume with dynamic susceptibility contrast-enhanced MR imaging. Radiology.

[22] Meier P, Zierler KL (1954). On the theory of the indicator-dilution method for measurement of blood flow and volume. J Appl Physiol.

[23] Farin G, Farin G (2002). The Bernstein form of a Bézier curve. Curves and surfaces for CAGD.

[24] Okell TW, Chappell MA, Schulz UG, Jezzard P (2012). A kinetic model for vessel-encoded dynamic angiography with arterial spin labeling. Magn Reson Med.

[25] Meijs M, Christensen S, Lansberg MG, Albers GW, Calamante F (2016). Analysis of perfusion MRI in stroke: to deconvolve, or not to deconvolve. Magn Reson Med.

[26] Knutsson L, Lindgren E, Ahlgren A, van Osch MJP, Bloch KM, Surova Y, Ståhlberg F, van Westen D, Wirestam R (2014). Dynamic susceptibility contrast MRI with a prebolus contrast agent administration design for improved absolute quantification of perfusion. Magn Reson Med.

[27] Peruzzo D, Castellaro M, Pillonetto G, Bertoldo A (2017). Stable spline deconvolution for dynamic susceptibility contrast MRI. Mag Reson Med.

[28] Pizzolato M, Boutelier T, Deriche R (2017). Perfusion deconvolution in DSC-MRI with dispersion-compliant bases. Med Image Anal.

[29] Giacalone M, Frindel C, Robini M, Cervenansky F, Grenier E, Rousseau D (2017). Robustness of spatio-temporal regularization in perfusion MRI deconvolution: An application to acute ischemic stroke. Magn Reson Med.

[30] McKinley R, Hung F, Wiest R, Liebeskind DS, Scalzo F (2018). A Machine Learning Approach to perfusion imaging with dynamic susceptibility contrast MR. Front Neurol.

[31] Fan AP, Jahanian H, Holdsworth SJ, Zaharchuk G (2016). Comparison of cerebral blood flow measurement with [15O]-water positron emission tomography and arterial spin labeling magnetic resonance imaging: a systematic review. J Cereb Blood Flow Metab.

