# Supplementary figures and images for: Non-parametric deconvolution using Bézier curves for quantification of cerebral perfusion in dynamic susceptibility contrast MRI
Source: MAGMA. 2022 Jan 13;35(5):791–804. doi: 10.1007/s10334-021-00995-0 (PMC9463354; doi:10.1007/s10334-021-00995-0)

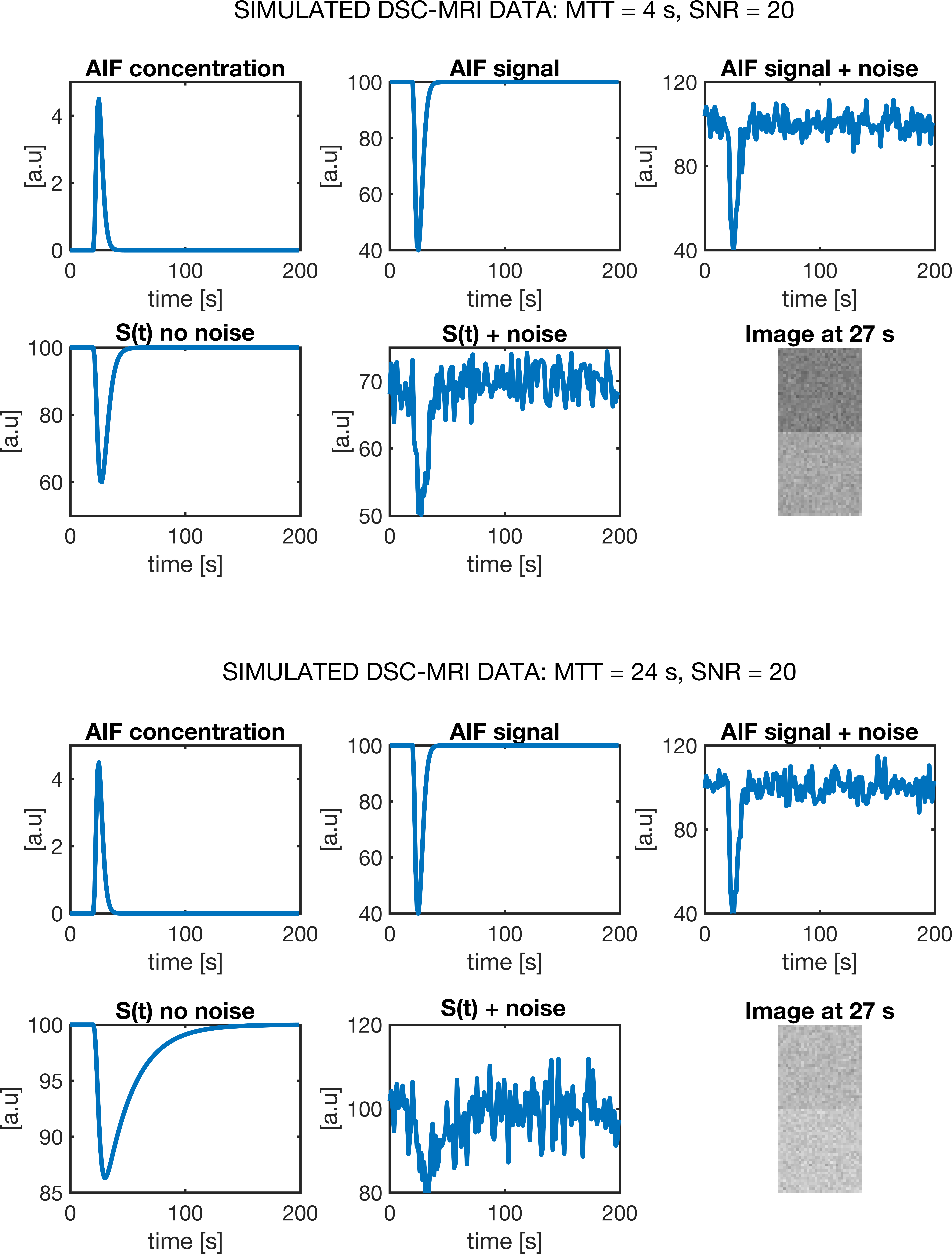

Supplement: Supplementary file 1 — Supplementary file1 (TIF 10654 KB) Simulated DSC-MRI data examples for MTT = 4s (top) and MTT = 24 s (bottom). The images (labeled “Image at 27 s”) show the simulated typical gray matter and white matter pixels. The data were generated at SNR = 20 [file 10334_2021_995_MOESM1_ESM.tif]
