# Supplementary material for: Non-parametric deconvolution using Bézier curves for quantification of cerebral perfusion in dynamic susceptibility contrast MRI
Source: MAGMA. 2022 Jan 13;35(5):791–804. doi: 10.1007/s10334-021-00995-0 (PMC9463354; doi:10.1007/s10334-021-00995-0)
Supplement: Supplementary file 4 — Supplementary file4 (PDF) Accuracy of delay and dispersion parameter estimation [file 10334_2021_995_MOESM4_ESM.pdf]

Online Resource 4: Accuracy of delay and dispersion parameter estimation

Results for the accuracy of delay and dispersion parameter estimation with BzD are shown in Table S1. The delay parameter δ was most accurately estimated in the absence of dispersion and the dispersion parameter p was generally best determined in the absence of delay. The accuracy of these parameters generally decreased with increasing degree of the corresponding effect. When both delay and dispersion were present, the delay parameter was generally overestimated while the dispersion parameter was generally underestimated. Except for p in the case of low delay and dispersion, the sum of the parameters $(\delta + p)$ was more accurately determined than the individual parameters. The observed trends in the accuracy of estimating the delay and dispersion parameters suggest that the deconvolution algorithm tends to merge delay and dispersion into a single AIF-distorting effect. Consequently, adequate correction for the delay and dispersion effect, from a perfusion parameter perspective, does not necessarily imply that delay and dispersion effects have been individually correctly addressed. Indeed, delay correction in the presence of dispersion without delay was found to improve CBF estimation (see Online Resource 5).

Table S1. Relative errors in the estimation of the delay parameter (δ) and one of the dispersion parameters (p) under different simulated scenarios. The results shown were generated with CBV 4%, CBF 60 ml/100g/min and SNR 100.

Parameter	Percentage error		
	Low	Medium	High
Delay (no dispersion) δ	(7 ± 13) %	(1 ± 6) %	(-1 ± 6) %
Dispersion (no delay) p	(90 ± 40)%	(30 ± 30)%	$(-4 \quad \pm 9)$ %
Delay (with dispersion) δ	(70 ± 70) %	(50 ± 30) %	(20 ± 40)%
Dispersion (with delay) p	(-3 ± 50)%	(-70 ± 50)%	(-30 ± 60)%
Dispersion and delay $\delta + p$	(30 ± 40)%	(-11 ± 12) %	(-2 ± 9) %
